# The Genomic Landscape of *TP53* and p53 Annotated High Grade Ovarian Serous Carcinomas from a Defined Founder Population Associated with Patient Outcome

**DOI:** 10.1371/journal.pone.0045484

**Published:** 2012-09-20

**Authors:** Paulina M. Wojnarowicz, Kathleen Klein Oros, Michael C. J. Quinn, Suzanna L. Arcand, Karen Gambaro, Jason Madore, Ashley H. Birch, Manon de Ladurantaye, Kurosh Rahimi, Diane M. Provencher, Anne-Marie Mes-Masson, Celia M. T. Greenwood, Patricia N. Tonin

**Affiliations:** 1 Department of Human Genetics, McGill University, Montreal, Quebec, Canada; 2 Division of Clinical Epidemiology and Segal Cancer Centre, Lady Davis Research Institute, Jewish General Hospital, Montreal, Quebec, Canada; 3 Centre de recherche du Centre hospitalier de l’Université de Montréal (CRCHUM), Institut du Cancer de Montréal, Montreal, Quebec, Canada; 4 The Research Institute of the McGill University Health Centre (MUHC), Montreal, Quebec, Canada; 5 Department of Pathology, Centre Hospitalier de l’Université de Montréal (CHUM), Montreal, Quebec, Canada; 6 Division of Gynecologic Oncology, Université de Montréal, Montreal, Quebec, Canada; 7 Department of Medicine, Université de Montréal, Montreal, Quebec, Canada; 8 Department of Oncology, McGill University, Montreal, Quebec, Canada; 9 Department of Epidemiology, Biostatistics and Occupational Health, McGill University, Montreal, Quebec, Canada; 10 Department of Medicine, McGill University, Montreal, Quebec, Canada; Univesity of Texas Southwestern Medical Center at Dallas, United States of America

## Abstract

High-grade ovarian serous carcinomas (HGSC) are characterized by *TP53* mutations and non-random patterns of chromosomal anomalies, where the nature of the *TP53* mutation may correlate with clinical outcome. However, the frequency of common somatic genomic events occurring in HGSCs from demographically defined populations has not been explored. Whole genome SNP array, and *TP53* mutation, gene and protein expression analyses were assessed in 87 confirmed HGSC samples with clinical correlates from French Canadians, a population exhibiting strong founder effects, and results were compared with independent reports describing similar analyses from unselected populations. *TP53* mutations were identified in 91% of HGSCs. Anomalies observed in more than 50% of *TP53* mutation-positive HGSCs involved gains of 3q, 8q and 20q, and losses of 4q, 5q, 6q, 8p, 13q, 16q, 17p, 17q, 22q and Xp. Nearly 400 regions of non-overlapping amplification or deletion were identified, where 178 amplifications and 98 deletions involved known genes. The subgroup expressing mutant p53 protein exhibited significantly prolonged overall and disease-free survival as compared with the p53 protein null subgroup. Interestingly, a comparative analysis of genomic landscapes revealed a significant enrichment of gains involving 1q, 8q, and 12p intervals in the subgroup expressing mutant p53 protein as compared with the p53 protein null subgroup. Although the findings show that the frequency of *TP53* mutations and the genomic landscapes observed in French Canadian samples were similar to those reported for samples from unselected populations, there were differences in the magnitude of global gains/losses of specific chromosomal arms and in the spectrum of amplifications and deletions involving focal regions in individual samples. The findings from our comparative genomic analyses also support the notion that there may be biological differences between HGSCs that could be related to the nature of the *TP53* mutation.

## Introduction

Ovarian cancer is the fifth leading cause of cancer-related death in the developed world. This is largely attributed to the high recurrence rates and poor overall survival of the 90% of cancers that are epithelial ovarian cancers [1], [2]. This group of cancers has been classified based on histopathology and tumour grade, where high-grade ovarian serous carcinomas (HGSC) represent the most frequently reported malignancy [2], [3]. The etiology of HGSCs is unknown, though about 10% have been associated with inherited mutations in the *BRCA1*/*BRCA2* cancer susceptibility genes [4]. HGSCs frequently exhibit somatic *TP53* mutations that have been reported in more than 90% of cases [5]. Recently it has been suggested that HGSCs with *TP53* null mutations (detected by the absence of immunostaining for p53 protein) are associated with an unfavourable outcome [6]. These findings not only have important implications in clinical management but may reflect biological differences in HGSCs based on the consequences of the nature of the *TP53* mutation in the cancer sample as also suggested by recent gene expression profiling of ovarian cancer cases, which showed differences in transcriptome profiles in samples stratified based on the absence or the nature of the *TP53* mutation [7]. The heterogeneity of HGSC is also reflected by the high frequency of chromosomal anomalies involving copy number gains and losses, loss of heterozygosity (LOH), and complex intrachromosomal rearrangements [8], [9]. Cytogenetic, array comparative genomic hybridization, LOH and most recently SNP array technologies have shown that specific genomic anomalies are enriched in HGSCs [8]–[12]. For example, LOH of a chromosome 17 contig [13]–[16] is common in HGSC and this has been attributed to the disruption of *TP53* (17p13.1) [13], [17], [18], *BRCA1* (17q12) (in some cases) [19], [20], and most recently other candidate tumour suppressor genes [21]–[27]. Though common genomic anomalies have emerged implicating specific molecular pathways that define subsets of HGSCs, significant heterogeneity continues to confound studies aimed at identifying and characterizing genomic targets important in disease etiology [8], [9].

Few studies to date have described genomic anomalies occurring in HGSCs from genetically well-defined populations. A study by the Australian Ovarian Cancer Study (AOCS) group found fewer anomalies across the genome in Japanese versus Australian-derived HGSCs though their findings were not significant [9]. We recently reported an unusual excess of copy number neutral regions of homozygosity involving chromosome 3 in borderline ovarian serous tumours [28]. In our study the tumour samples were from French Canadians of Quebec (Canada) [28], a population that has a unique genetic demography [29]. This population exhibits strong founder effects, attributed to the geographic isolation and expansion of French settlers between 1608–1759, and is known for its contribution to medical genetics [29]. A recent study of lymphocyte DNA reported that subpopulations of French Canadians varied in their genomic structure and degrees of relatedness, and contained significantly more copy number neutral regions of homozygosity than samples from Europeans [30]. While regions of homozygosity may have an impact on complex diseases, including cancer [31], their significance is unknown. It is possible that during cancer development these genomic regions of homozygosity may be subject to chromosomal rearrangements that may contribute to shaping the genomic landscape of HGSCs in French Canadians.

Here we describe, for the first time, whole genome SNP array, *TP53* mutation, and *TP53* gene and protein expression analyses in HGSC samples with defined clinical correlates from the demographically unique French Canadian population, and compare our findings with independent reports describing similar analyses of cancer specimens from unselected populations. We report that the overall landscape of genomic anomalies largely overlaps those reported by two recent SNP array studies of HGSCs by the AOCS group [9] and The Cancer Genome Atlas (TCGA) Research Network [8], though the magnitude of losses and gains differed for specific chromosomal regions, including loci involved in genomic amplifications and homozygous deletions. We also report that the *TP53* mutation frequency is similar to that reported in independent studies [5], [8] and that p53 protein null mutation cases were associated with poorer overall and disease-free survival, comparable to a recent report [6]. We also explored the possibility that differences in *TP53* mutation type might be reflected in genomic landscapes, and report the unique finding that samples expressing mutant p53 protein, which were the cases associated with prolonged overall and disease-free survival, showed significant copy number gains of specific chromosomal regions, relative to *TP53* mutation-positive, p53 protein null samples.

## Results

### Defining the HGSC Sample set for Comparative Analyses

Whole genome SNP array, *TP53*, *BRAF* and *KRAS* mutation, and *TP53* expression analyses were performed on 99 primary HGSC samples from chemotherapy naïve cases, selected from a tumour database. Paraffin embedded ovarian cancer tissue samples from all 99 samples were subsequently independently reviewed by a gynecologic pathologist. Pathology review and tumour database re-annotation revealed that 87 of the 99 cases analyzed unambiguously retained their HGSC subtype classification, and were from chemotherapy naïve patients (Table 1, Table S1).

**Table 1:** *TP53* mutation spectrum and global chromosomal anomalies assessed by SNP array analyses in 87 HGSC verified cases.

		Chromosomal Anomalies	
Number of samples	*TP53* mutation type	Present	Absent
48	Missense	48	0
12	frameshift-stop	12	0
10	splice	10	0
8	nonsense	8	0
1	in-frame insertion	1	0
8	wild-type	3	5

See Table S1 for Complete Description of *TP53* Mutations.

### *TP53*, *BRAF* and *KRAS* Mutation Spectrum in HGSC

*TP53* mutation analysis was performed to determine the frequency and spectrum of mutations in HGSC samples from French Canadian women. Mutations were identified in 79 of 87 (91%) HGSCs (Table 1). Most of the variants identified (Table S1) are suspected to affect p53 function and have been reported in various cancer samples by the IARC *TP53* Database [32]. About 61% (48 of 79) of mutation-positive HGSCs had a missense mutation that is predicted to affect either the DNA binding (n = 47) or oligomerization (n = 1) domain of the encoded p53 protein (Table S1). The remaining 31 (39%) mutation-positive HGSCs harbour variants that are predicted to affect the ability to encode a protein as a consequence of the introduction of a nonsense codon and/or alteration in reading frame due to frameshift, splice or in-frame deletion (Table S1). Both the spectrum and frequency of *TP53* mutations observed in our HGSCs were consistent with independent reports where comprehensive DNA sequencing or exomic sequencing (as in TCGA study) was performed [5], [8].

Somatic mutations in the oncogenes *KRAS* or *BRAF* usually occur as mutually exclusive events in epithelial ovarian cancer, have been reported in borderline ovarian serous carcinomas or low-grade ovarian serous carcinomas, and occur at a low frequency in HGSCs [33]. None of the HGSC cases were found to have *KRAS* or *BRAF* mutations and the paucity of mutations in these genes is consistent with independent reports [8], [33].

### *TP53* Gene and Protein Expression in HGSC and Association with Disease-free and Overall Survival

**Figure 1:**
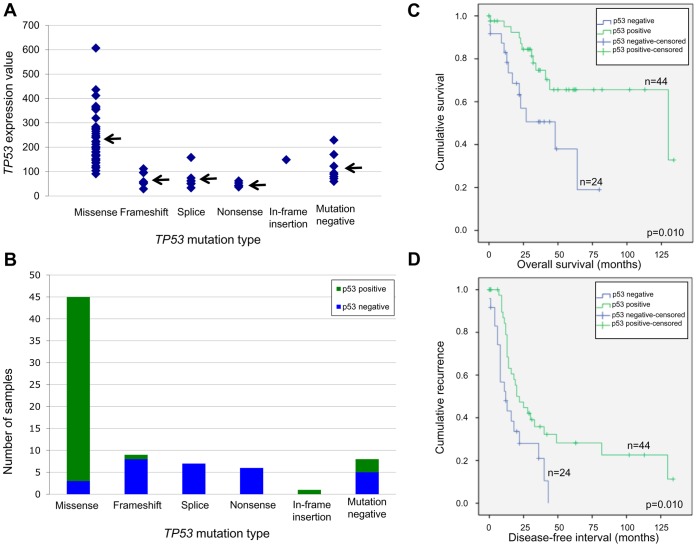
*TP53* gene expression, protein expression and Kaplan-Meier survival curve analyses in HGSC cases. Ziplex® custom array-derived *TP53* gene expression data for 76 of the 87 HGSC cases, grouped according to *TP53* mutation type (A). The mean *TP53* gene expression values for missense (n = 45), frameshift (n = 9), splice (n = 7), nonsense (n = 6), in-frame insertion (n = 1) and mutation-negative (n = 8) HGSC cases are 232.2, 67.6, 70.1, 46.6, 148.9, and 114.5, respectively, as indicated by arrows. Immunohistochemistry analysis of 76 HGSC cores, arrayed on a tissue microarray, grouped according to *TP53* mutation type (B). Positive p53 immunoreactivity was observed for 42/45 missense, 1/9 frameshift, 0/7 splice, 0/6 nonsense, 1/1 in-frame insertion, and 3/8 mutation-negative HGSC cases. Kaplan-Meier survival curve analysis of *TP53* mutation-positive HGSC cases for overall survival, in months, of patients positive for p53 staining (n = 44) compared to patients negative for p53 staining (n = 24) (C). Kaplan-Meier survival curve analysis of *TP53* mutation-positive HGSC cases for disease-free interval, in months, of patients positive for p53 staining (n = 44) compared to patients negative for p53 staining (n = 24) (D). Indicated p-values were derived from Mantel-Cox, log-rank tests.

*TP53* gene and protein expression was assessed in HGSCs to determine if the nature of the mutation affected mRNA expression levels by *TP53* gene expression array analysis or nuclear p53 protein staining by immunohistochemistry of a tissue array. To examine these effects we focused our analyses on the 76 (out of 87) samples for which deriving both gene and protein expression data was possible. *TP53* expression showed a significantly higher average level of expression in missense mutation-containing samples (mean = 232.2) than samples harbouring frameshift-stop (mean = 67.6), splice (mean = 70.1) or nonsense (mean = 46.6) mutations (p<0.001) (Figure 1A). Immunoreactivity with an anti-p53 antibody was observed in 47 of the 76 (62%) samples using a tissue array (Figure 1B). The majority of samples showed uniform, diffuse staining throughout the core. Missense mutation-positive samples were more likely to express mutant p53 protein (42 of 45, 93%) than samples with other types of mutations (2 of 23, 9%). Not surprisingly, all samples with nonsense mutations exhibited no p53 staining (p53 protein null). Interestingly, three of the eight *TP53* mutation-negative samples expressed p53 protein (see Figure 1B), suggesting the possibility of missed mutations in our sequencing analysis or the deregulation of other pathways that result in the stabilization of p53 protein [34].

Overall and disease-free survival were assessed for the *TP53* mutation-positive HGSCs as recent evidence suggests improved survival in cases expressing mutant p53 protein [6]. Kaplan-Meier survival curve analysis found that the subgroup expressing mutant p53 protein had significantly longer overall survival (p = 0.010) and disease-free interval (p = 0.010) than the p53 protein null subgroup (Figure 1C, D).

### High-density Genome-wide Genotyping of HGSCs

**Figure 2:**
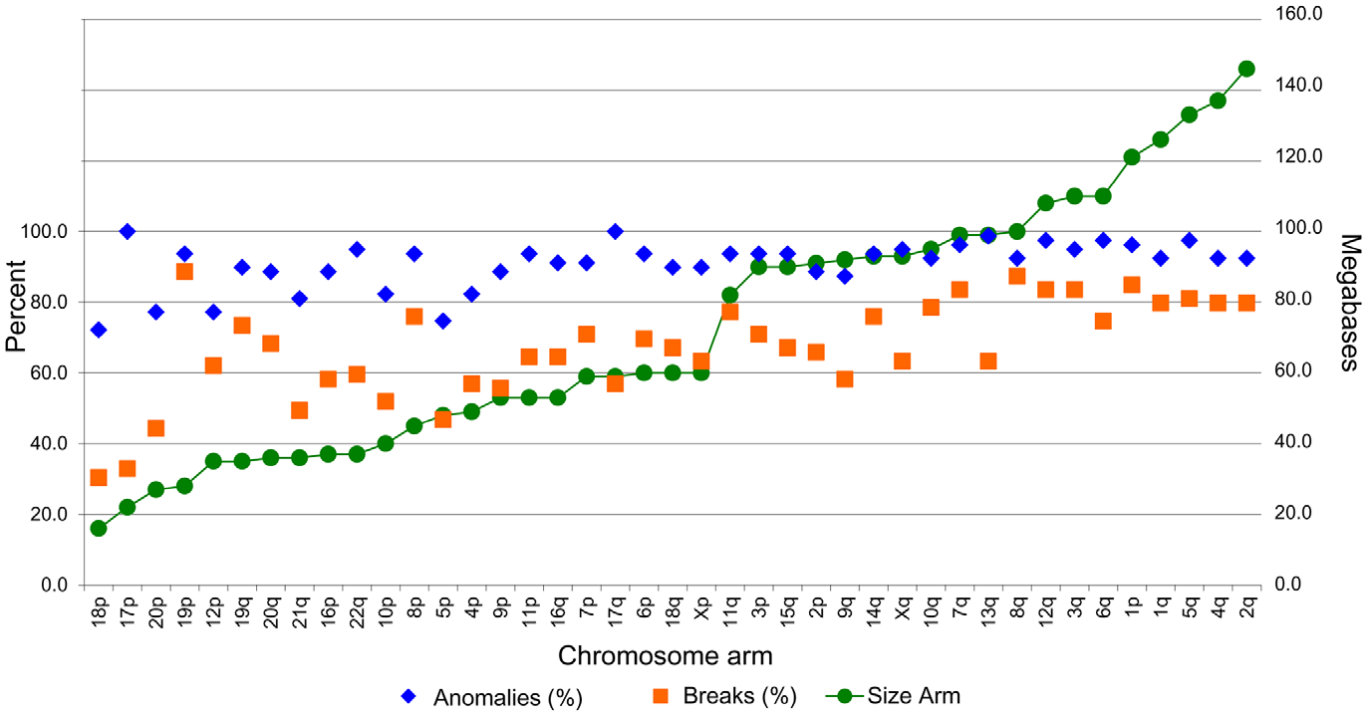
Chromosomal anomalies and breaks across the genome of *TP53* mutation-positive HGSC cases. Percentage of the 79 *TP53* mutation-positive HGSCs with chromosomal anomalies, including copy number alteration and allelic imbalance, and breaks occurring per chromosome arm, as inferred by reviewing image files created by the Genome Viewer module in BeadStudio Data Analysis software v2.2.22. The chromosome arms are arranged according to their size in megabases, left to right from smallest to largest.

Chromosomal anomalies were assessed using high-density SNP arrays. Allelic imbalance, copy number differences and intrachromosomal breaks were inferred visually using the Genome Viewer module of the BeadStudio software. Chromosomal anomalies were evident in all *TP53* mutation-positive HGSCs (Table 1) but only in three of the eight *TP53* mutation-negative HGSCs (Table 1). We focused our analysis on the 79 *TP53* mutation-positive HGSCs as there were too few *TP53* mutation-negative samples for comparative analyses and only a small subset (3 of 8) exhibited evidence of chromosomal anomalies (Table 1). The presence of an anomaly on any chromosomal arm varied between 72% and 100%, and the presence of any intrachromosomal break (on any chromosomal arm), as suggested by a deviation in Log R ratio of adjacent flanking markers, varied from 30.4% to 88.6% (Figure 2). The patterns of anomalies observed were not necessarily related to the overall size of the chromosome or chromosomal arm (Figure 2). Although, chromosome 17 anomalies were detectable in 100% of samples, both chromosomal arms exhibited the lowest proportion of intrachromosomal breaks, suggesting whole chromosome loss (and/or duplication). In contrast, 19p anomalies, which were observed in 94% of all samples, exhibited the highest percentage of at least one intrachromosomal arm break (89%). A high proportion of intrachromosomal breaks relative to allelic imbalances were also observed with 8q (87%), 1p (85%), 12q (84%), 7q (84%) and 3q (84%).

**Figure 3:**
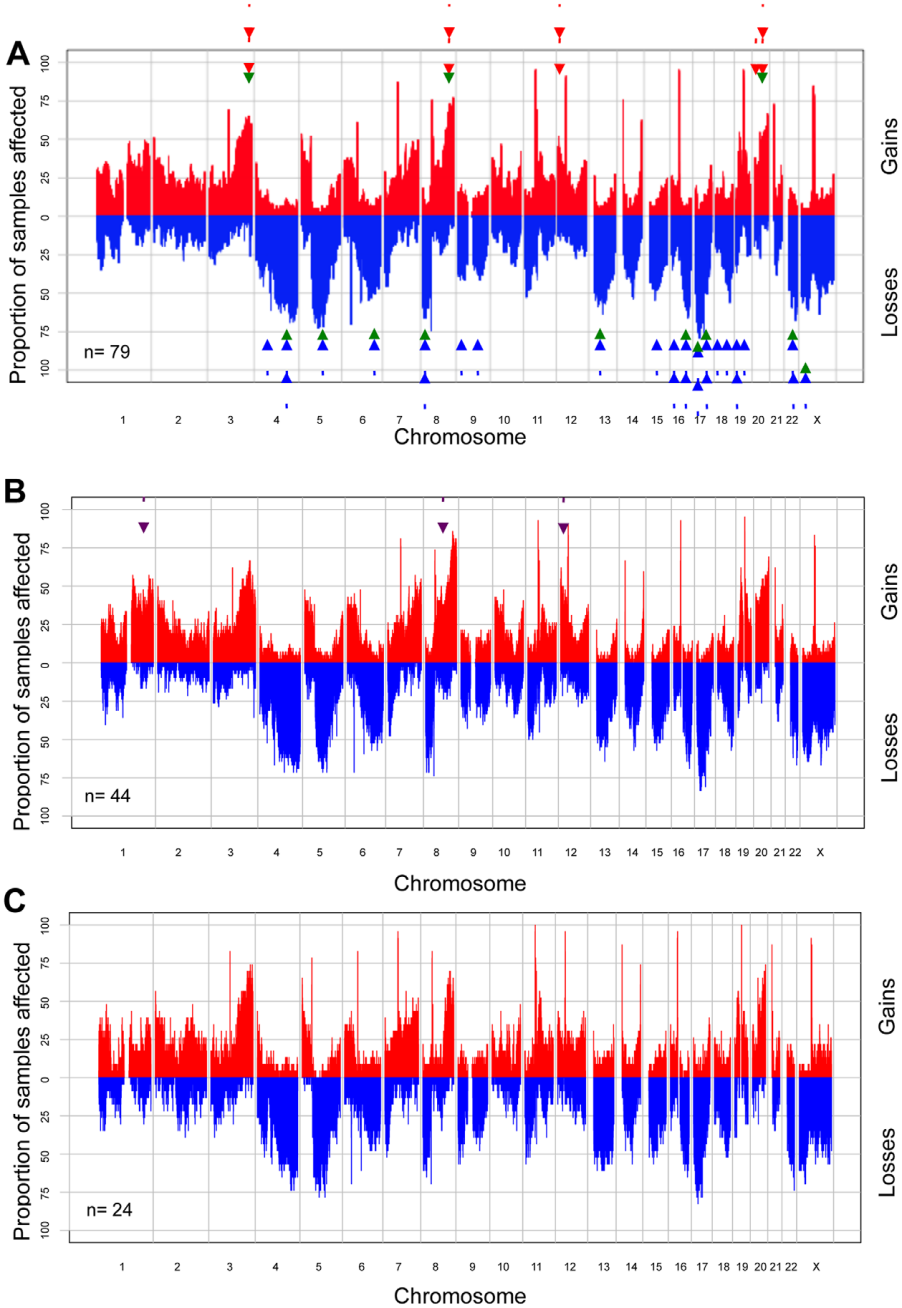
Global regions of gain and loss in *TP53* mutation-positive HGSC cases. Global regions of gain and loss in the 79 *TP53* mutation-positive HGSC cases, as determined by GenoCNA analyses (A). Regions of gain and loss occurring in >50% of the 79 HGSC cases are indicated by green arrowheads. Regions of gain and loss occurring in >50% of the TCGA samples [8] and in >39% of the AOCS samples [9] are indicated by solid and dashed arrows, respectively (red colour indicates gain, blue colour indicates loss). Global regions of gain and loss in the 44 HGSC cases expressing p53 mutant protein (B). Global regions of gain and loss in the 24 p53 protein null HGSCs (C). Regions found to be significantly gained in the cases expressing p53 mutant protein as compared to the p53 protein null cases by GenoCNA analyses include 1q, 8q and 12p, and are indicated by purple arrows.

**Figure 4:**
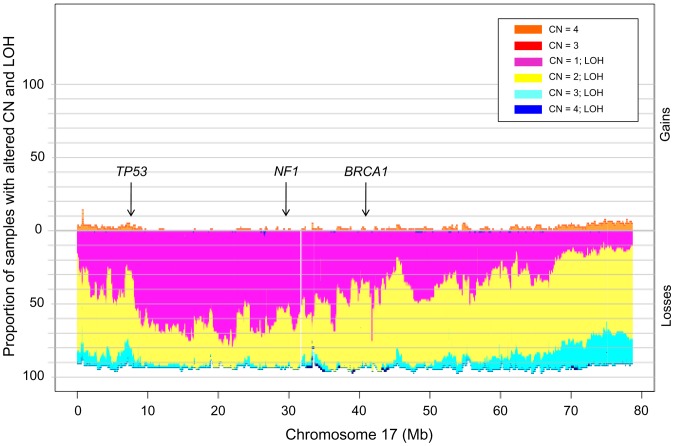
Chromosome 17 copy number alterations in *TP53* mutation-positive HGSC cases. GenoCNA analysis of Chromosome 17 copy number alterations in the 79 *TP53* mutation-positive HGSC cases. The genomic loci of the genes *TP53*, *NF1*, and *BRCA1* are indicated with arrows. CN, copy number; LOH, loss of heterozygosity.

To investigate the frequency of common anomalies, we applied GenoCNA analyses to the *TP53* mutation-positive HGSCs. Copy number gains occurring in greater than 50% of samples involved 3q, 8q and 20q, whereas losses occurring in greater than 50% of samples involved 4q, 5q, 6q, 8p, 13q, 16q, 17p, 17q, 22q and Xp (Figure 3A). The chromosome 17 profile was striking: though *TP53* is located at 17p13.1, nearly all samples exhibited evidence of LOH across the entire chromosome, although copy number varied (Figure 4), as described in independent reports [13], [14], [16], [20], [35]. The genomic landscape of our samples was very similar to that reported in the analysis of 398 mostly HGSCs by the AOCS group [9] (Figure 3A). With few exceptions, the majority of gains and losses exceeding 39% in the AOCS study overlapped regions exhibiting similar anomalies in our samples (Figure 3A). Noticeable differences were the higher frequencies of losses of portions of 5q, 6q and 13q, which exceeded 50% in our samples but occurred less often (<39%) in the AOCS samples [9]. It was more difficult to directly compare our results with the analysis of 489 HGSC samples reported by TCGA as a combined genomic landscape was not reported [8]. However, the frequency of anomalies exceeding 50% involved more chromosomal arms in the TCGA samples than that found in our study (or as compared with the AOCS study), and with few exceptions the chromosomal regions involved overlapped those exhibiting anomalies in our sample set (Figure 3A). All chromosome arm losses occurring in at least 50% of the TCGA samples exhibited losses in at least 25% of our samples with the exception of 18p.

Manual examination of the data for each *TP53* mutation-positive sample identified 398 non-overlapping regions of amplification or homozygous deletion, where 178 amplifications involved known genes and 98 deletions were intragenic (Tables S2–S6). Most of these genetic anomalies were observed in individual samples. The regions of homozygous deletion overlapped regions of loss in 15–95% of samples (Table S2, Table S3). The regions of homozygous deletion identified in our samples overlapped only a fraction of those reported as focal regions of loss in independent studies (Table S2, Table S3, Table S5). For example, only 11 regions of focal loss in the TCGA study and 23 such regions in the AOCS study overlapped the 43 homozygous deletions that affected intragenic coding regions identified in our samples (Table S2) [8], [9]. Similarly, 12 and 23 of the 55 homozygous deletions identified in intronic regions were also identified by TCGA and AOCS studies, respectively (Table S3) [8], [9]. Twelve intragenic regions were found to overlap regions identified in both the TCGA and AOCS studies (Table 2) [8], [9]. Intergenic deletions were also observed and exceeded the number of deletions that are predicted to disrupt a gene (Tables S2–S4).

**Table 2:** 

Table 2A. Amplicons identified in the group of 79 *TP53* mutation-positive HGSC cases that overlap focal regions of gain in TCGA and AOCS studies							
Cytoband	Coordinates	Number of HGSCs with amplification	Number of genes mapping to amplicon	Genes*	p53 status of samples with amplification	% of HGSCs with gain ofthe region	Region overlaps global region of gain/loss identified in TCGA and AOCS studies
1p35.1-1p34.2	chr1:33008238-41528143	2	48	*MYCL1, SNIP1, BMP8A, HEYL, PABPC4*	+/−	25	
1q21.2-1q23.1	chr1:147844664-155604287	4	111	*ECM1, CTSS, HDGF, MCL1, GOLPH3L, HORMAD1*	+/−	35	
1q41-1q43	chr1:221535355-238714517	6	131	*WNT9A, ARF1, FMN2*	+/−	35	
1q43-1q44	chr1:240528827-242941460	2	12	*AKT3, SDCCAG8, ADSS*	+	30	
2p23.2-2p23.1	chr2:28440573-30468387	1	3	*FOSL2, PLB1, LBH*	+	30	
2p21	chr2:43910094-43974350	1	3	*ABCG5, ABCG8, LRPPRC*	+	20	
2q13-2q14.1	chr2:113658240-114686122	1	13	*PAX8, ACTR3, FOXD4L1*	–	15	
3q26.1-3q26.32	chr3:161617980-177762913	10	55	*MECOM, SKIL, CLDN11*	+/−	50	<
3q28-3q29	chr3:193553281-194082030	1	2	*FGF12, MB21D2*	–	45	<
5p15.33	chr5:869095-3398843	3	22	*TERT, NKD2, BRD9*	+	45	
6p22.3-6p22.2	chr6:18081947-24054846	4	12	*TPMT, SOX4, PRL*	+/−	30	
6p21.1	chr6:42454334-42741634	1	2	*TRERF1, UBR2*	+	25	
7q35	chr7:144488779-147219701	1	3	*CNTNAP2, MIR548I4, MIR548F4*	+	35	
8p12-8q11.1	chr8:37337810-47653281	9	45	*PROSC, FGFR1, ADAM9*	+/−	20	
8q11.22-8q12.1	chr8:51211670-58216011	3	29	*SOX17, RPS20, PENK, PLAG1*	+	15	<
8q24.13-8q24.3	chr8:124635537-145748936	14	122	*MYC, MAFA, PVT1, MIR1208*	+/−	50	<
10p15.3-10p15.1	chr10:300796-6530227	3	42	*KLF6, NET1, GDI2*	+	25	
10q22.2-10q23.1	chr10:75124035-85266181	4	52	*KCNMA1, VDAC2, NRG3*	+/−	15	
11q13.2-11q14.1	chr11:68511689-84849803	10	122	*ALG8, NUMA1, CCND1, CTTN, THRSP, RSF1*	+/−	25	
12p13.2-12p13.1	chr12:11814971-12699342	1	11	*DUSP16, BCL2L14, ETV6*	+	30	
12p12.3-12q12	chr12:16991152-38614568	16	79	*KRAS, CASC1, PIK3C2G, LYRM5*	+/−	25	
12q12-12q13.11	chr12:40539586-47134988	6	41	*HDAC7, VDR, NELL2*	+/−	15	
19p13.2-19p13.11	chr19:7553709-19137359	8	269	*JUNB, ICAM1, FBN3, NOTCH3, CACNA1A*	+/−	30	
19p12	chr19:20229532-21633219	2	14	*ZNF430, ZNF626, ZNF737*	+	15	
19p12-19q13.11	chr19:22730358-39491269	27	47	*CCNE1, POP4, PDCD5*	+/−	30	
19q13.12-19q13.2	chr19:41514434-46504853	8	139	*NFKBIB, MAP4K1, EGLN2, SYCN, PAK4, PAF1*	+/−	25	
20q11.1-20q11.21	chr20:28134044-30617934	2	34	*ID1, TPX2, BCL2L1, FOXS1, MYLK2*	+	15	<

Table 2B. Homozygous deletions identified in the group of 79 *TP53* mutation-positive HGSC cases that overlap focal regions of loss in TCGA and AOCS studies							
Cytoband	Coordinates	Number of HGSCs with deletion	Number of genes mapping to deletion	Genes	p53 status of samples with deletion	% of HGSCs with loss of the region	Region overlaps global region of gain/loss identified in TCGA and AOCS studies
3p26.1	chr3:6,626,929-6,629,060	8	1	*AF279782-intronic deletion*	+	45	
4q24	chr4:107,058,467-107,097,080	1	1	*NPNT*	+	65	<
4q25	chr4:108,285,187-108,288,489	1	1	*DKK2-intronic deletion*	+	70	<
5q21.3	chr5:108,323,379-108,492,133	1	1	*FER*	+	70	
6q16.1	chr6:94,019,483-94,034,629	1	1	*EPHA7*	+	50	
6q26	chr6:162,687,672-162,806,676	1	1	*PARK2*	Na	65	
8p23.2	chr8:391,493-521,421	1	2	*FBXO25, C8orf42*	–	60	<
8p21.2	chr8:25,468,655-25,470,302	1	1	*PPP2R2A-intronic deletion*	+	60	<
9p24.1	chr9:3,248,448-3,691,912	1	1	*RFX3*	–	55	
17q11.2	chr17:26,545,913-26,807,370	1	5	*NF1, OMG, EVI2B, EVI2A, RAB11FIP4*	+	90	<
21q21.1	chr21:18,249,338-18,250,201	9	1	*CHODL-intronic deletion*	+/−	50	
21q21.1	chr21:19,046,840-19,329,426	1	1	*PPIAL3-intronic deletion*	–	45	

HGSC, high-grade ovarian serous carcinoma; +, expression of p53 mutant protein; −, p53 protein null; +/−, samples with amplification/deletion include both p53-positive and negative cases; na, no p53 data for the sample.

* Subset of genes is listed for amplicons with >3 genes, the full list of genes is presented in Table S5

The discrete regions of genomic amplification overlapped regions of gain in 2–55% of the *TP53* mutation-positive samples (Table S5). As observed with homozygous deletions, only a fraction of the regions of amplification in our samples overlapped focal regions of gain reported in independent studies (Table 2). For example, only 35 focal regions of gain from the TCGA study and 78 such regions from the AOCS study overlapped the 178 gene-containing amplifications identified in our *TP53* mutation-positive samples (Table S5) [8], [9]. Only 27 gene-containing amplifications overlap focal regions of gain reported by TCGA and AOCS studies (Table 2) [8], [9].

As a genotyping analysis on patient matched normal samples was not feasible in our study, we integrated our findings with the database of copy number variants, which facilitated interpretation of results (http://projects.tcag.ca/variation/). For example, the homozygous deletion at 8p11.23 found in 16 of 79 (20.3%) of samples has been reported in this database of common genomic variants (Figure 3A). However there were examples of homozygous deletions and amplifications involving known genes in our sample set that do not overlap regions involving known copy number variants. One of these regions contained *EPHA7* (6q16.1), found deleted in one sample in our study. This region overlapped regions of focal loss reported in the TCGA and AOCS studies (Table 2) [8], [9].

### Comparative SNP Array Analyses of *TP53* Mutation-positive Samples Parsed by p53 Immunoreactivity

**Figure 5:**
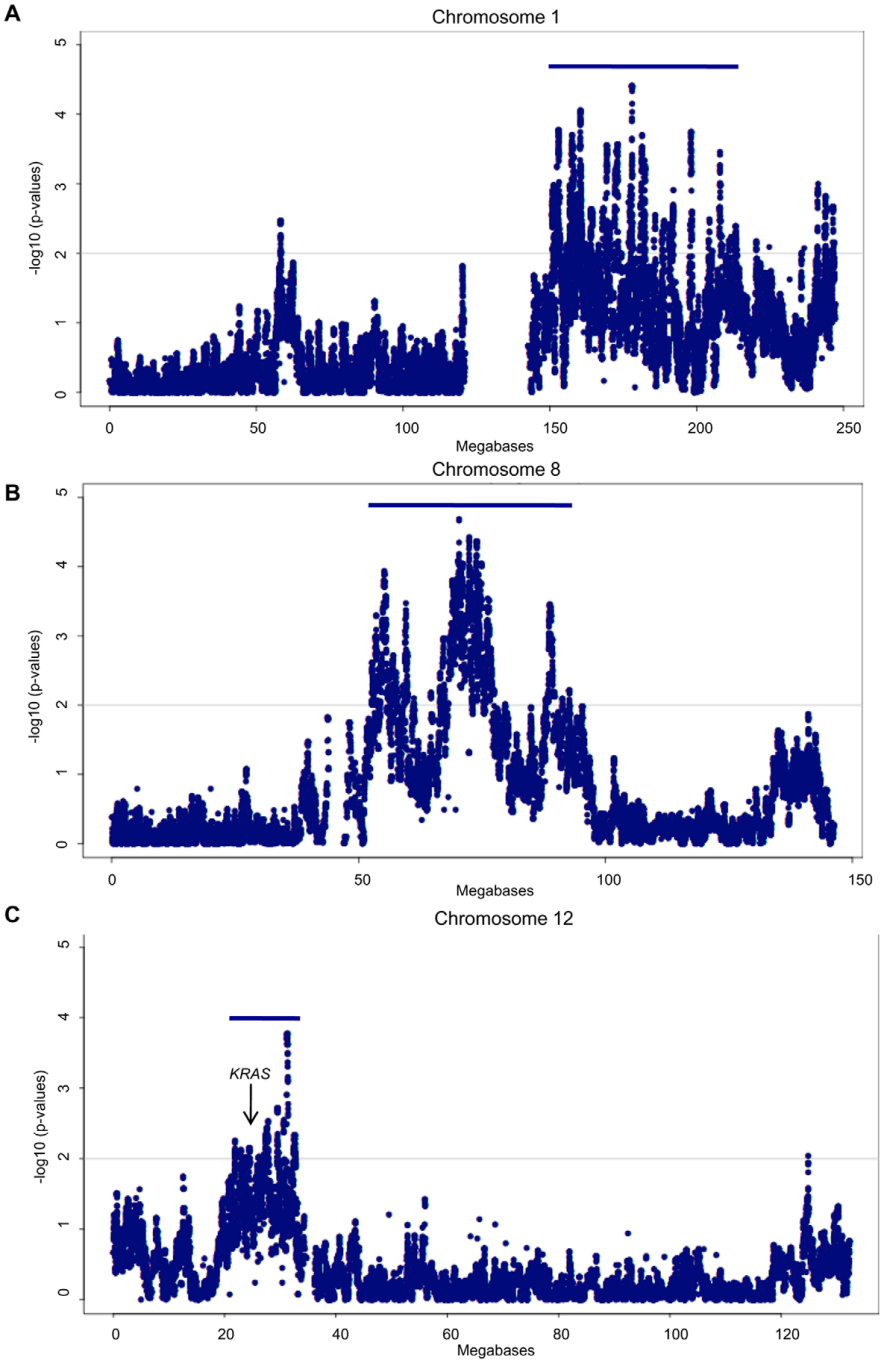
GenoCNA analysis comparing HGSC cases based on p53 immunoreactivity. GenoCNA analysis comparing the mean copy number, on a per SNP basis, of the 44 samples expressing p53 mutant protein to that of the 24 p53 protein null HGSC cases for chromosomes 1 (A), 8 (B) and 12 (C), which contain regions significantly gained in the HGSC cases expressing p53 mutant protein as compared to the p53 protein null HGSC cases (see Figure 3B, C). Points on the plot represent –log10 of p-values from t-tests comparing the means in the two groups. Blue bars indicate regions of significant gain.

The differences observed in disease-free and overall survival based on p53-immunoreactivity suggested the possibility of biological differences related to the nature of the *TP53* mutation [6] and thus we questioned if they could also be reflected in the genomic landscapes. We re-evaluated global patterns of anomalies using GenoCNA where the samples were parsed according to p53-immunoreactivity. Not surprisingly, the genomic landscapes of the subgroup expressing mutant p53 protein and that of the p53 protein null subgroup were remarkably similar (Figure 3B, C). However, visual inspection suggested that gain of some regions of 1q, 8q and 12p were less evident in the p53 protein null subgroup (Figure 3B, C). A statistical analysis of copy number variation between the two groups revealed significant (p>0.001) gains of specific regions of 1q, 8q and 12p intervals in the subgroup expressing mutant p53 protein as compared with the p53 protein null subgroup (Figure 5, Figure 3B, C). Although neither the AOCS and TCGA studies performed a similar comparative analysis of their HGSC samples, 8q and 12p were reported as chromosome arms that showed common gain in the AOCS study [9], and 1q, 8q, and 12p were reported as chromosome arms that showed gain in greater than 50% of the TCGA samples [8]. In our analysis, gain of 8q intervals was found in at least 50% of samples, while 1q and 12p gain occurred in less than 50% of samples (Figure 3A).

## Discussion

Our mutation analysis of *TP53, KRAS* and *BRAF*, and SNP genotyping analyses suggest that the most common genetic and genomic anomalies arising in HGSC from French Canadian women were similar to those found in HGSCs from unselected populations. In particular, the genomic landscape of common gains and losses was remarkably similar to that reported by AOCS [9] and TCGA [8] groups. For example, the genes also found amplified in our study include *MECOM* at 3q26, which is thought to be a driver of amplification in some HGSCs [36] and *KRAS* at 12p12.3 (Table 2). Interestingly,*CSMD3* and *CDK12*, found “significantly” mutated (2.8–6.0%) in HGSC by the TCGA, map to regions of amplification identified in our study [8]. The tumour suppressor gene *NF1*, found deleted in one of our samples, was also reported as focally deleted in both the TCGA and AOCS studies [8], [9]. *NF1* was also one of nine genes (including *TP53*) found “significantly” mutated (∼3.6%) by TCGA [8]. *NF1* defects may be emerging as a low frequency, but recurrent event in HGSC [8], [9], [23]. Interestingly, the gene *EPHA7*, mentioned above, has recently been implicated as tumour suppressor gene and proposed as a targeted region of deletion in lymphomas [37]. Although certain focal amplification and deletions were found in common with all three studies, the majority of these events appeared to be unique to particular samples. The genes targeted by common events may reflect convergent pathways that are commonly deregulated in HGSC.

Differences in the genomic landscapes reflected in the magnitude of gains/losses observed in our samples as compared with AOCS and TCGA studies, could be a reflection of the methods used to assess global genomic anomalies among the studies. However, other factors may have influenced these findings. For example, the AOCS study also included samples that were not HGSCs and did not report on *TP53* mutation status or p53 immunoreactivity of their samples, and thus could have included *TP53* mutation-negative samples in their analyses, and the TCGA study did not exclude *TP53* mutation-negative cases from their analyses. As our findings show, chromosomal anomalies were less evident in *TP53* mutation-negative HGSCs. However, it is unlikely that the inclusion of such cases would have significantly affected the interpretation of their results, as they would have accounted for less than 10% of all samples analyzed.

Differences in somatic mutation frequencies of specific loci have been reported in tumours from ethnically defined populations. The most well documented example involves the frequency of activating mutations in the *EGFR* oncogene and copy number differences of the *EGFR* locus for certain types of lung and breast carcinomas [38]–[43]. A recent study comparing lung adenocarcinomas found differences in both copy number and mutation frequencies in samples from two ethnically different groups, which included a significantly higher rate of copy number gains of 16p13.13 and 16p13.11 intervals in East Asian samples, while copy number losses of 19p13.3 and 19p13.11 intervals occurred at a higher rate in Western European samples [44]. The AOCS study found fewer anomalies across the genome in Japanese versus Australian-derived HGSCs, though the differences were not reported and the finding was not significant [9]. Thus although our results demonstrate that the most common anomalies overlap those found in cancers from unselected populations, we cannot exclude the possibility that the genomic landscape observed in our HGSCs were not influenced by the genetic background of the French Canadian population examined [28]–[31]. Assessing genetic and genomic events in demographically defined populations may have implications in clinical management, as shown by the ethnic-specific differences in responsiveness to treatment with EGFR tyrosine kinase inhibitors in specific cancer therapies [39], [40].

The significant enrichment of gains involving specific 1q, 8q and 12p intervals in HGSCs expressing mutant p53 protein as compared with p53 protein null samples suggest that there are biological differences between these two groups. This is supported by the differences in disease-free and overall survival between these two *TP53* mutation-positive groups. Studies that have evaluated the correlation of p53-immunoreactivity with clinical outcome have produced conflicting results, and this may have been due to analyses that included different histological subtypes of ovarian cancer as well as incomplete assessment of *TP53* mutation status, where mutation status was inferred by immunohistochemistry or where mutation screening was limited to specific exons [6], [45]–[50]. Indeed our results are consistent with a recent report associating p53 protein null HGSCs, which were also assessed for *TP53* mutation, with poorer overall outcome [6]. Furthermore, another independent study has shown that p53 null mutations were more common in ovarian cancer cases with distant metastasis [51]. Our results are interesting in light of the observation made by the AOCS study that there were no strong correlations between copy number variations involving specific loci and clinical outcome, though weak associations between overall survival and copy number gains of both 3q13 and 19q12 intervals or losses of both 17q12 and 22q intervals were found [9]. However this group did not report the nature of *TP53* mutation in samples analyzed [9]. The TCGA study also did not appear to report on any associations with the nature of the *TP53* mutations [8]. An earlier, independent study reported differences in the transcriptomes of serous ovarian carcinomas grouped according to the presence, absence and nature of *TP53* mutation [7]. In this study *LMNA* expression was found to be significantly higher in samples with missense *TP53* mutations relative to samples with null mutations, and this gene maps within an interval on 1q showing gain in our HGSCs expressing mutant p53 protein [7]. Furthermore, low LMNA protein expression was recently found associated with increased disease recurrence in colon cancer, a pattern consistent with our findings relating p53-immunoreactivity status and outcome of HGSC patients [52].

The regions on 1q and 8q implicate a large number of candidates as targets of amplification. The estimated 10 Mb region of gain on 12p appeared more discrete in size facilitating a survey of candidates implicated. While this interval also contains numerous genes (Table S7), it also harbours the well known oncogene *KRAS* [53], and as mentioned above the *KRAS* locus has also been reported as a focal region of gain by TCGA and AOCS [8], [9]. Amplification of the *KRAS* locus has been suggested as an alternative mechanism for deregulating *KRAS*-implicated pathways in HGSC given that oncogene-activating point mutations in this gene are infrequently observed [8], [9].

Our findings relating clinical outcome to the nature of the *TP53* mutation are not surprising given that alteration of p53 function, as a consequence of missense mutation, can result in a range of activities but not necessarily abolition of the transcriptional activity exhibited by wild-type protein [54]. For example, *in vitro* mutagenesis assays of codon 175 found different effects depending on the amino acid that replaced the wild-type arginine, ranging from wild-type function to mediating only cell cycle arrest [55]. Thus while *TP53* mutations may be a near ubiquitous feature of HGSC, our findings suggest that HGSCs show differences in copy number gains according to the p53-immunoreativity status of the sample, these differences may in part be attributed to the effects of altered p53 function.

In conclusion, our genomic analyses of *TP53* mutation-positive HGSCs from French Canadians identified global anomalies in common with HGSCs examined from populations not selected for ethnicity. This finding strengthens the notion that the genomic intervals involved likely contain genes that are biologically relevant to the development of HGSC. Our findings are the first to define genomic landscapes based on mutant p53-immunoreactivity and clinical outcome and this warrants replication in independent samples from the French Canadian and other populations. In particular, it would be interesting to investigate the genomic landscapes of the TCGA samples based on mutant p53 immunoreactivity and how they relate to the HGSC subtypes identified based various large-scale molecular analyses [8], [56]. The association with clinical outcome warrants further investigation of the genes associated with the chromosomal regions showing gains in the cases expressing mutant p53 protein, as this may allow for patient stratification and ultimately improve disease management.

## Materials and Methods

### Ethics Statement

The study was performed in accordance with the Declaration of Helsinki. Written informed consent was obtained from all participants involved in this study. The study is in compliance with the Helsinki declaration, and has been granted ethical approval by the respective Research Ethics Boards of the Centre hospitalier de l’Université de Montréal-Hôpital Notre-Dame and The McGill University Health Centre.

### Clinical Specimens and Clinical Correlates

Tumour samples were selected from a tumour database that were collected and banked with informed written consent from participants undergoing surgeries performed within the Division of Gynecologic Oncology at the Centre hospitalier de l’Université de Montréal-Hôpital Notre-Dame, from 1992 to 2010. Clinical features such as disease stage, and tumour characteristics such as grade and histopathological subtype, were assigned by a gynecologist-oncologist and gynecologic-pathologist, respectively, according to the criteria established by the International Federation of Gynecology and Obstetrics. Information about overall survival (rounded to the nearest month), defined from the time of surgery until patient death, and disease-free interval (rounded to the nearest month), defined from the time of surgery to the time of doubling of the upper normal limit of the serum cancer antigen marker CA-125 or the detection of a new lesion by ultrasound or CT-scan imaging, were extracted from the Système d’Archivage des Données en Oncologie (SARDO). Overall survival was defined from the time of diagnosis to ovarian cancer-related death. In cases where death was not related to ovarian cancer the data was censored in the statistical analyses of overall survival.

### Nucleic Acid Extraction

DNA was extracted from fresh frozen tumour specimens as described previously [57]. Total RNA was extracted with TRIzol™ reagent (Invitrogen Canada Inc., Burlington, ON) from fresh frozen tumours as described previously [58]. RNA quality was assessed by gel electrophoresis or 2100 Bioanalyzer analysis using the RNA 6000 Nano LabChip kit (Agilent Technologies, Mississauga, ON).

### Gene Mutation Analysis

Mutation analysis of tumour DNA samples was designed to detect variants in the protein coding exons 2 to 11, and adjacent splice sites of *TP53*, as well as the common mutations in exon 2 of *KRAS* and exons 11 and 15 of *BRAF.* Mutation analysis of *TP53* and *KRAS* was performed using PCR-based assays followed by sequencing of both genomic strands using the 3730XL DNA Analyzer system (Applied Biosystems, Carlsbad, California) at the McGill University and Genome Quebec Innovation Center (Montreal, QC, Canada) as previously described [28], [59], [60]. Mutation analysis of *BRAF* was performed using PCR-based assays followed by single-strand conformation polymorphism (SSCP) analysis as previously described [59], or sequencing of both genomic strands as above and previously described [28]. Primer sequences for each assay were as reported previously [28], [59], [60]. Sequence chromatograms were compared with NCBI reference sequence (RefSeq) reported in GenBank: *NM_000546.4* (*TP53*), *NM_004985.3* (*KRAS*) and *NM_004333.4* (*BRAF*), and the genomic structures available from the February 2009 GRCh37/hg19 assembly of the human reference genome. Sequence variants were compared with those reported in the SNP Database (www.ncbi.nlm.nih.gov/SNP). In addition, *TP53* variants were evaluated based on information in the International Agency for Research on Cancer (IARC) *TP53* Database (www-p53.iarc.fr).

### Gene Expression Analysis of *TP53*

Expression of *TP53* was determined for 87 high-grade ovarian serous carcinoma samples. Gene expression was assessed using a custom Ziplex® Research System gene expression array platform (Axela Inc., Toronto, Canada). The array, probe design, and methodology have been described in detail [61]. Gene expression values were evaluated by ANOVA to assess associations with *TP53* mutation types. Analyses were performed with SPSS software version 16.0 (SPSS Inc., Chicago, IL USA), and p-values less than 0.05 were considered significant.

### Immunohistochemistry Analysis of p53

Immunohistochemistry analysis of p53 was performed using a tissue array containing 281 cores that included cores from 78 of the 87 HGSCs analyzed for *TP53* gene expression analysis. Of the 78 cores, 70 cores were from the 79 *TP53* mutation-positive HGSCs. The cores (0.6 mm diameter) of each tissue sample, based on the review of a hematoxylin-eosin stained slide, were punched and arrayed into a paraffin block. The tissue array was sectioned, stained with hematoxylin-eosin and pathology reviewed again to confirm content. Immunohistochemistry was preformed using the Ventana Benchmark XT automated immuno-stainer (Ventana Medical Systems, Tucson, AZ, USA). Briefly, the ultraView Universal DAB Detection Kit (#760–500) was used with 60-minute antigen retrieval in Cell Conditioning buffer #2 (active ingredient is citrate buffer). Primary antibody (p53 DO-1: sc-126) was diluted at 1∶200 and incubated for 28 minutes at 37°C. Slides were scanned on a ScanScope XT Digital Scanner (Aperio Technologies, Vista, CA, USA). Results were assessed by two independent observers and scored for presence or absence of staining, with absence reflecting no detectable staining (Inter-observer correlation coefficient  = 0.907). No score was given for two of the 78 cores due to poor quality. These two cores were from *TP53* mutation-positive HGSCs. The inter-observer correlation coefficient was calculated using SPSS software version 16.0 (SPSS Inc., Chicago, IL USA), where the minimum threshold was 0.7.

The relationship between positive or negative p53 staining and overall survival or disease-free interval as defined above was evaluated using Kaplan-Meier survival curve analysis coupled to the Mantel-Cox log-rank test. Analyses were performed with SPSS software version 16.0 (SPSS Inc., Chicago, IL USA), and p-values less than 0.05 were considered significant.

### High-density Genotyping

Genome-wide chromosomal anomalies in 99 ovarian tumours were inferred using the Infinium™ genotyping technology with Illumina’s Human610-Quad Genotyping BeadChip (Illumina, San Diego, CA, USA). This BeadChip assays 620,901 markers, where over 560,000 are SNPs with an average spacing of 4.7 Kb per marker (median spacing is 2.7 kb). Both genotyping, using 750 ng of DNA from frozen tumours, and scanning, using the BeadArray™ Reader (Illumina, San Diego, CA, USA), were performed at the McGill University and Genome Quebec Innovation Centre (Montreal, QC, Canada). All samples had call rates (the percentage of valid genotype calls) within the range of 0.914 and 0.999 (average 0.992).

Genotyping analysis was performed using the Genome Viewer module in BeadStudio Data Analysis software v2.2.22 (Illumina, San Diego, CA, USA.). The software aligns genotyping data for each marker with genomic map coordinates based on the March 2006 NCBI36/hg18 (Build 36.1) assembly of the human reference sequence (genome.ucsc.edu/cgi-bin/hgGateway). An image file was created for inferring genomic rearrangements based on the allele frequency and copy number (Log R ratios) for each marker assayed. LOH was inferred by B allele frequency, where values that deviate from 0.5 (less than 0.4 and greater than 0.6) indicate allelic imbalance when reviewed for a series of adjacently mapped markers. Breakpoints were inferred based on deviation of allele frequencies relative to that of adjacently mapped markers. Log R ratios deviating from 0 suggest copy gain or loss. Homozygous deletions were inferred based on Log R ratios ≤−2 for at least three adjacently mapped markers, and sizes were estimated based on the location of nearest flanking markers with Log R ratios above -2. Amplifications were inferred based on a mean Log R ratio ≥0.5 for at least 40 adjacently mapped markers. Means were calculated in 40 marker windows and gaps of <100 markers were tolerated. End points had a minimum Log R ratio of 0.4 and a gap of <40 markers to the next marker with a Log R ratio of 0.4. Amplifications were consolidated per sample by visual inspection of the regions graphically, and regions >15 Mb were removed. Regions of overlap between samples were determined and sizes were estimated based on the location of the nearest flanking markers or chromosome telomere.

Normalized SNP intensity files were also analyzed by GenoCNA [62]. This software uses a hidden Markov model containing nine different tumour states, including loss of 1 or 2 copies, copy number neutral loss of heterozygosity (LOH), and five different gain states allowing for different patterns of allele retention. This model explicitly allows for normal tissue contamination in the samples. Graphs show the percentage of the samples with gains or losses based on the GenoCNA inference, where the percentage is calculated in expectation, using the average of the probabilities of relevant states at each marker. Using the copy numbers estimated by GenoCNA for each individual, we calculated the mean and standard deviation of the copy numbers, at each SNP, for the samples expressing mutant p53 protein and those that were p53 protein null. Copy number estimates ranged between 0 (complete loss) and 4 (a gain of two extra copies). A t-test was performed comparing the means of copy number differences of the two *TP53* mutation-positive groups defined by p53 immunoreactivity. The data was visualized by comparing –log10 p-values per position of markers along the chromosome. The –log10 p-values greater than 2 were considered significant.

## Supporting Information

Table S1 **HGSC samples used in global genotyping analyses.** Legend: TOV, malignant ovarian tumour; D, sample from tumour on right ovary; G, sample from tumour on left ovary; EP, sample from tumour in omentum; M, sample from metastasis; n/a left-right designation of sample unknown; GX, grade of sample unknown; IHC, immunohistochemistry; EX, exon; IVS, intervening sequence; CCOC, clear cell ovarian carcinoma; OSC, serous ovarian carcinoma; LGOSC, low-grade serous ovarian carcinoma; HGSC, high-grade ovarian serous carcinoma. Note, that no *BRAF* mutations were identified in any sample (n = 99).
(XLS)

Table S2 **Homozygous deletions affecting intragenic coding regions identified in** ***TP53*** **mutation positive HGSC samples.** Legend: HGSC, high-grade ovarian serous carcinoma; CHR, chromosome; CNV copy number variation. Blue font indicates genes identified as somatically mutated in TCGA, 2011.
(XLS)

Table S3 **Homozygous deletions affecting intragenic intronic regions identified in** ***TP53*** **mutation positive HGSC samples.** Legend: HGSC, high-grade ovarian serous carcinoma; CHR, chromosome; CNV copy number variation. Blue font indicates genes identified as somatically mutated in TCGA, 2011
(XLS)

Table S4 **Homozygous deletions affecting gene-flanking regions identified in** ***TP53*** **mutation positive HGSC samples.** Legend: HGSC, high-grade ovarian serous carcinoma; CHR, chromosome; CNV copy number variation.
(XLS)

Table S5 **Amplicons identified in** ***TP53*** **mutation positive HGSC samples, containing genes.** Legend: HGSC, high-grade ovarian serous carcinoma; CHR, chromosome; CNV copy number variation.
(XLS)

Table S6 **Amplicons identified in** ***TP53*** **mutation positive HGSC samples, containing no genes.** Legend: HGSC, high-grade ovarian serous carcinoma; CHR, chromosome; CNV copy number variation.
(XLS)

Table S7 A) Number of RefSeq genes mapping to approximate regions of gain in p53-positive HGSC cases as compared to p53-negative HGSC cases. B) Lists of RefSeq genes mapping to approximate regions of gain in p53-positive HGSC cases as compared to p53-negative HGSC cases. RefSeq genes extracted from UCSC table browser based on the March 2006 NCBI36/hg18 (Build 36.1) assembly of the human reference sequence (genome.ucsc.edu).
(XLS)
